# Efficacy and safety of upadacitinib in the treatment of rheumatoid arthritis: a systematic review and meta-analysis

**DOI:** 10.3389/fimmu.2026.1806265

**Published:** 2026-05-20

**Authors:** Yi-Heng Xu, Dan Liu, Meng-Rui Zhang, Yi-Jing Zhang, Ming Li, Qing-Rui Yang

**Affiliations:** 1 Department of Rheumatology and Immunology, Shandong Provincial Hospital Affiliated to Shandong First Medical University (Shandong Provincial Hospital), Jinan, China; 2 Department of Gastroenterology in Health Building, Shandong Provincial Hospital Affiliated to Shandong First Medical University (Shandong Provincial Hospital), Jinan, China

**Keywords:** JAK inhibitor, meta-analysis, randomized controlled trial, rheumatoid arthritis, upadacitinib

## Abstract

**Background:**

Rheumatoid arthritis (RA) is a chronic systemic autoimmune disease characterized by persistent synovial inflammation, leading to progressive joint destruction and functional impairment. Although conventional synthetic disease-modifying antirheumatic drugs (csDMARDs), particularly methotrexate, remain the first-line therapy, a substantial proportion of patients exhibit inadequate responses and require escalation to biologic or targeted synthetic therapies. Upadacitinib, a selective Janus kinase 1 (JAK1) inhibitor administered orally, has demonstrated promising efficacy in phase III trials; however, a comprehensive evaluation of its dose-dependent efficacy and safety across different patient populations remains lacking.

**Methods:**

This systematic review and meta-analysis was conducted in accordance with the PRISMA guidelines. Electronic databases, including PubMed, Web of Science, and Embase, were systematically searched from inception to July 2025 for randomized controlled trials evaluating upadacitinib in patients with RA. The primary outcome was the proportion of patients achieving an American College of Rheumatology 20% improvement (ACR20) response at 12 weeks. Secondary outcomes included safety endpoints such as overall adverse events, serious infections, herpes zoster, and laboratory abnormalities. Meta-analyses were performed using RevMan 5.4, with fixed- or random-effects models applied based on heterogeneity. The study protocol was registered with PROSPERO under identifier CRD420251134541.

**Results:**

Nine randomized controlled trials involving 5,237 participants were included. Both 15 mg and 30 mg once-daily upadacitinib significantly improved ACR20 response rates at 12 weeks compared with control treatments (15 mg: OR=4.09, 95% CI 3.51–4.76; 30 mg: OR=3.61, 95% CI 2.88–4.52; both P < 0.00001). No significant difference in efficacy was observed between the two dosage regimens (OR=1.00, P=0.98). In terms of safety, upadacitinib was associated with an increased risk of adverse events, with a dose-dependent trend observed (15 mg: OR=1.30; 30 mg: OR=1.42). The incidence of specific adverse events, including serious infections, herpes zoster, and elevated liver enzymes, was higher in the 30 mg group.

**Conclusion:**

Upadacitinib demonstrates superior efficacy compared with control treatments in patients with RA, with both 15 mg and 30 mg doses providing comparable clinical benefits. However, the higher dose is associated with an increased risk of adverse events, suggesting that the 15 mg regimen may offer a more favorable benefit–risk balance. Further studies are warranted to evaluate long-term outcomes and safety in specific patient populations.

**Systematic review registration:**

https://www.crd.york.ac.uk/prospero/display_record.php?RecordID=1134541, identifier CRD420251134541.

## 1 Introduction

Rheumatoid arthritis (RA) is a chronic autoimmune disease characterized by persistent synovial inflammation that leads to joint pain, swelling, and progressive structural damage (1). Without timely and standardized treatment, ongoing inflammation results in cartilage destruction, loss of function, and long-term disability, substantially impairing quality of life (2). The global prevalence of RA is estimated at 0.5%–1.0% (3), with a prevalence of approximately 0.42% in China. As disease duration increases, so do the risks of joint deformity, reduced work capacity, and healthcare burden, underscoring the need for effective therapeutic strategies. Current treatment aims to rapidly control inflammation and achieve remission or low disease activity to prevent structural damage (4). Conventional synthetic disease-modifying antirheumatic drugs (csDMARDs), particularly methotrexate, are recommended as first-line therapy (5); however, 30%–50% of patients exhibit inadequate responses (6). In such cases, treatment escalation to biologic DMARDs (bDMARDs) or targeted synthetic DMARDs (tsDMARDs) is required (7, 8). Nevertheless, biologic therapies are limited by injectable administration, immunogenicity, and infection risk, and some patients are intolerant to combination regimens (6). These limitations highlight the need for effective oral therapies with favorable safety profiles.

Upadacitinib is a highly selective Janus kinase 1 (JAK1) inhibitor that blocks key pro-inflammatory cytokine signaling pathways, including IL-6, IL-12/23, and interferon-mediated pathways (9), thereby reducing synovial inflammation and bone destruction (10). Since its approval by the U.S. Food and Drug Administration in 2019, its efficacy has been demonstrated in multiple phase III trials. For example, a multinational study reported that 71.6% of patients receiving upadacitinib 15 mg plus csDMARDs achieved an ACR20 response at 12 weeks, compared with 31.4% in the placebo group, with significant differences observed as early as week 1 (11). In patients with inadequate response to methotrexate, upadacitinib showed greater reductions in DAS28-CRP and improvements in physical function and quality of life compared with adalimumab (12, 13). In treatment-naïve patients, upadacitinib monotherapy achieved higher remission rates than methotrexate (14, 15). Furthermore, in patients failing prior biologic therapy, comparative efficacy data between upadacitinib and alternative mechanism-of-action agents (e.g., abatacept: a modulator targeting T-cell co-stimulation) provide critical evidence to inform clinical decision-making (16).

In terms of safety, commonly reported adverse events include upper respiratory tract infections, herpes zoster, and laboratory abnormalities such as neutropenia and elevated liver enzymes. The risks of serious infections and venous thromboembolism appear comparable to those observed with tumor necrosis factor inhibitors (13), and prior meta-analyses have not shown a significant increase in malignancy risk (17). However, potential safety concerns remain in high-risk populations, including elderly patients, smokers, and those with increased cardiovascular risk (18). Despite these advances, several important gaps remain. Many existing meta-analyses do not include the most recent randomized controlled trials and lack detailed stratification by dosage, treatment duration, and patient subgroups. In addition, long-term outcomes, particularly radiographic progression beyond 24 weeks, remain insufficiently characterized. Evidence regarding special populations, such as elderly patients and those with hepatic impairment, is also limited, restricting individualized treatment strategies. In light of these limitations, the present meta-analysis aims to comprehensively evaluate the dose–response relationship of upadacitinib. Specifically, this study compares the efficacy of 15 mg and 30 mg regimens in achieving ACR20 responses, assesses dose-dependent safety profiles, explores differences between monotherapy and combination therapy, and evaluates regional variations in treatment outcomes.

Among JAK inhibitors, including tofacitinib, baricitinib, and deucravacitinib, differences in selectivity may influence clinical outcomes (19). Upadacitinib, as a selective JAK1 inhibitor, offers a targeted mechanism that may improve efficacy while reducing off-target effects. Emerging evidence suggests that JAK selectivity may also affect safety profiles, particularly with respect to hematologic toxicity, infection risk, and cardiovascular events (20). Given these characteristics, a focused evaluation of upadacitinib is clinically warranted.

## 2 Materials and methods

### 2.1 Inclusion and exclusion criteria

#### 2.1.1 Types of included studies

This meta-analysis exclusively incorporated publicly available randomized controlled trials (RCTs). Due to resource constraints, only studies published in English and Chinese were included. This restriction may introduce language bias and limit the generalizability of the findings.

#### 2.1.2 Participants

Eligible patients were required to meet all of the following criteria: a confirmed diagnosis of moderate-to-severe active rheumatoid arthritis (RA) based on either the 1987 ACR/EULAR classification criteria or the 2010 revised criteria, an age of 18 years or older, and no restrictions with respect to ethnicity or nationality. Patients were excluded if they had concomitant autoimmune disorders, such as systemic lupus erythematosus or ankylosing spondylitis, or if they presented with significant organ dysfunction, defined as hepatic impairment of Child–Pugh class B or higher, or renal impairment indicated by a creatinine clearance of less than 30 mL/min. This exclusion strategy was implemented to minimize potential confounding effects on the assessment of both efficacy and safety outcomes.

#### 2.1.3 Interventions

The intervention protocols were defined according to the original study designs. In the experimental arms, patients received oral upadacitinib at daily doses of either 15 mg or 30 mg. Participants in the control arms were allocated to comparator regimens consisting of either placebo monotherapy or placebo administered in combination with conventional synthetic disease-modifying antirheumatic drugs (csDMARDs), such as methotrexate. For safety outcome analyses, comparisons between the experimental group and each control subgroup were conducted separately and are presented individually in the corresponding forest plots.

All interventions were implemented in accordance with the original trial protocols, with a minimum treatment duration of 12 weeks. This dual-control design facilitates direct comparisons between upadacitinib-based regimens and established standard-of-care therapeutic approaches.

#### 2.1.4 Outcome measures

##### 2.1.4.1 Efficacy outcomes

The primary efficacy endpoint was the proportion of patients achieving an ACR20 response at 12 weeks. ACR20 was selected as the primary endpoint because it represented the most consistently reported outcome across the included studies, thereby allowing for robust cross-trial comparison. Other clinically relevant endpoints, including ACR50, ACR70, Disease Activity Score in 28 joints (DAS28), radiographic progression, and quality-of-life measures, were not incorporated into the primary analysis due to their inconsistent or limited reporting among the eligible trials.

An ACR20 response was defined as a ≥20% improvement from baseline in both tender joint count and swollen joint count, in addition to a ≥20% improvement in at least three of the following five parameters: patient-reported pain assessed by the visual analog scale (VAS), patient global assessment (PGA), physician global assessment (PhGA), physical function measured by the Health Assessment Questionnaire Disability Index (HAQ-DI), and an acute-phase reactant, either C-reactive protein (CRP) or erythrocyte sedimentation rate (ESR).

##### 2.1.4.2 Safety outcomes

This section defines the prespecified safety outcomes of clinical interest. Total adverse events (AEs) were defined as any untoward medical occurrences observed during the study period, regardless of their suspected relationship to the investigational drug. Infection-related events included severe infections, which were defined as those requiring hospitalization, administration of intravenous antibiotics, or considered life-threatening, such as pneumonia or sepsis. Herpes zoster was defined as clinically diagnosed or laboratory-confirmed reactivation of varicella–zoster virus.

Laboratory abnormalities were categorized according to clinically relevant thresholds, including hematologic abnormalities defined as a neutrophil count below 1.5 × 10^9^/L, hepatic abnormalities defined as alanine aminotransferase (ALT) or aspartate aminotransferase (AST) levels exceeding two times the upper limit of normal (ULN), and renal abnormalities defined as serum creatinine levels greater than 1.5 times the ULN. Thrombotic and cardiovascular events encompassed venous thromboembolism (VTE), including deep vein thrombosis and pulmonary embolism, as well as major adverse cardiovascular events (MACE), defined as stroke, myocardial infarction, or cardiovascular death. Malignancies were defined as pathologically confirmed solid tumors or hematologic cancers, with the exception of non-melanoma skin cancers. Death events were recorded as all-cause mortality, with an assessment of their potential relationship to the study treatment where applicable.

#### 2.1.5 Exclusion criteria

Studies were excluded if they met any of the predefined exclusion criteria. First, non-randomized study designs, including reviews, systematic reviews, case reports, and cohort studies, were excluded due to the inherent risk of selection bias associated with non-randomized evidence. Second, in cases where multiple publications reported results from the same clinical trial, only the most recent and most comprehensive dataset was retained, while duplicate publications were removed. Third, studies were excluded when data were inaccessible or incomplete, including situations in which full texts or abstracts could not be obtained, or when essential outcome data—such as ACR20 responder rates or adverse event frequencies—were not reported and could not be retrieved through correspondence with the study authors. Fourth, studies were excluded if they did not conform to the predefined intervention criteria, specifically those evaluating upadacitinib at dosages other than 15 mg or 30 mg once daily, or those with treatment durations shorter than 12 weeks. Finally, studies involving special populations, including pregnant women and pediatric patients, were excluded due to substantially different pharmacokinetic and safety profiles requiring separate analytical consideration.

### 2.2 Literature search strategy

#### 2.2.1 Search databases

A comprehensive literature search strategy was implemented by combining multiple English-language bibliographic databases with gray literature sources in order to maximize study retrieval and reduce the risk of publication bias. The primary electronic databases, searched from inception through July 2025, included PubMed, Web of Science, and Embase. To further enhance coverage, gray literature sources were systematically explored. These included pharmaceutical trial registries, particularly AbbVie’s Clinical Trials & Research portal (https://www.abbvie.com/clinical-trials.html), using a structured search strategy based on the terms (“upadacitinib” OR “ABT-494”) AND (“rheumatoid arthritis” OR RA) AND “randomized controlled trial”. In addition, dissertation databases such as ProQuest Dissertations & Theses Global and the China National Knowledge Infrastructure (CNKI) Master’s and Doctoral Dissertation Database were searched to identify unpublished academic research. Regulatory documents were retrieved from authoritative sources including Drugs@FDA, the European Medicines Agency (EMA) human medicines database, and ILAR research reports.

Finally, clinical trial registries and conference proceedings were screened, including ClinicalTrials.gov, the World Health Organization International Clinical Trials Registry Platform (WHO ICTRP), as well as abstracts from the European Alliance of Associations for Rheumatology (EULAR) Annual Meetings and the American College of Rheumatology (ACR) Annual Meetings from 2019 to 2025.

#### 2.2.2 Search terms

The combined search string for English databases (PubMed, Web of Science, Embase) was constructed as follows, combining controlled vocabulary (MeSH/Emtree) and free-text terms across three domains: intervention, condition, and study design dimensions to ensure comprehensive retrieval: (Upadacitinib OR “JAK inhibitor” OR “Janus kinase inhibitor” OR “JAK1 inhibitor”).

AND (“Rheumatoid Arthritis” OR RA).

AND (“Randomized Controlled Trial” OR RCT OR “Clinical Trial”).

Detailed database-specific search strategies (including PubMed and Embase) are provided in **Supplementary Material 1**. Both controlled vocabulary (MeSH in PubMed and Emtree in Embase) and free-text terms were used to ensure comprehensive retrieval.

### 2.3 Study screening and data extraction

#### 2.3.1 Study screening

A dual-reviewer, independent screening process with a structured disagreement-resolution mechanism was employed. Two reviewers, both with expertise in clinical rheumatology and/or evidence-based medicine, independently conducted a two-stage screening procedure. In the first stage, titles and abstracts were screened to exclude clearly ineligible studies, such as review articles and studies not focused on rheumatoid arthritis. In the second stage, the full texts of the remaining records were assessed in detail against the predefined inclusion and exclusion criteria. Any discrepancies between the two reviewers were resolved through consensus discussion. In cases where consensus could not be reached, a third senior reviewer with expertise in evidence-based medicine acted as an adjudicator and made the final decision. This structured and hierarchical approach was adopted to ensure an objective, transparent, and unbiased study selection process.

#### 2.3.2 Extraction of study data

A standardized data extraction form was developed to systematically collect relevant study information. Extracted data included fundamental study characteristics, such as the first author, country or region, year of publication, and trial registration number. Baseline participant characteristics were also recorded, including total sample size, group-specific sample sizes, age, sex distribution, disease duration, rheumatoid arthritis disease activity and prior treatment history, such as previous use of conventional synthetic disease-modifying antirheumatic drugs(csDMARDs) or biologic agents. In addition, detailed intervention information was extracted, including upadacitinib dosage, comparator treatment regimens, and overall treatment duration. Outcome data comprised both efficacy and safety endpoints, specifically the number of patients achieving ACR20 response and the incidence of each predefined adverse event category. Study quality-related variables were also collected, including the methods of randomization, allocation concealment, blinding procedures, attrition rates, and the handling of missing data. Following initial data extraction, an independent reviewer performed cross-validation of all extracted information to ensure accuracy and consistency of the dataset.

### 2.4 Quality assessment of included studies

For the included randomized controlled trials (RCTs), risk of bias was assessed using the Cochrane Risk of Bias tool (as implemented in RevMan 5.4), with evaluation performed across seven domains: selection bias arising from random sequence generation; selection bias due to allocation concealment; performance bias related to blinding of participants and investigators; detection bias concerning blinding of outcome assessors; attrition bias reflecting completeness of outcome data; reporting bias associated with selective outcome reporting; and other sources of bias, including potential funding-related influence or baseline imbalances between groups. Each domain was judged according to standardized criteria. A “low risk” rating was assigned when appropriate and clearly described methodological procedures were reported, such as computer-generated randomization sequences or centralized allocation concealment. A “high risk” rating was assigned when methods were clearly described but inherently prone to bias, such as allocation based on order of enrollment or absence of blinding. An “unclear risk” rating was used when methodological details were insufficiently reported, for example when only “random allocation” was stated without further specification of the randomization process. Two reviewers independently performed all risk of bias assessments. Any disagreements were resolved using the same consensus-based process applied during study selection, with arbitration by a third reviewer when necessary.

.

### 2.5 Statistical methods

The meta-analyses were carried out using RevMan 5.4 software as the analytical tool, with a two-tailed significance level set at α = 0.05. The specific methods are detailed as follows (21):

#### 2.5.1 Selection of effect measures

All outcome measures were dichotomous variables (e.g., “ACR20 response: yes/no”, “adverse event occurrence: yes/no”). The effect size was expressed as the OR with its 95%CI. An OR > 1 indicated a higher incidence of the outcome event in the experimental group than in the control group, while an OR < 1 indicated a lower incidence of the outcome event in the experimental group than in the control group. Odds ratios (ORs) were used as the effect measure for dichotomous outcomes, as they are the default metric in RevMan and allow consistency across studies. However, for relatively common outcomes such as ACR20, ORs may overestimate the effect size compared with risk ratios (RRs), and therefore the results should be interpreted with caution. A sensitivity analysis using RRs was not performed because the included studies did not consistently report the detailed data required for accurate conversion (e.g., complete event counts and denominators for all subgroup analyses). Conducting such analyses based on incomplete data may introduce bias and reduce the reliability of the results.

#### 2.5.2 Heterogeneity assessment

Between-study heterogeneity was assessed using the Chi-squared (χ^2^) test and the I^2^statistic, which quantifies the proportion of total variability attributable to heterogeneity rather than chance. A P value ≥ 0.10 in theχ^2^test together with an I^2^ value ≤ 50% was considered indicative of low heterogeneity. Under these conditions, a fixed-effects model was applied for effect size pooling, assuming a common underlying effect across studies, with study weights primarily determined by sample size and within-study variance.

In contrast, when P < 0.10 or I^2^ > 50%, substantial heterogeneity was considered to be present. In such cases, subgroup analyses and sensitivity analyses were first conducted to explore potential sources of heterogeneity. If heterogeneity could not be adequately explained, a random-effects model was adopted for meta-analysis, which assumes that true effect sizes vary across studies and incorporates both within-study variance and between-study heterogeneity into the weighting process.

#### 2.5.3 Sensitivity analysis

Sensitivity analyses employed a leave-one-out methodology, targeting two key outcomes: the 12-week ACR20 response rate comparing 15 mg versus 30 mg upadacitinib and overall adverse event incidence. For each iteration, individual studies were sequentially excluded, with effect sizes re-pooled and compared against the original model. Result robustness was confirmed when the pooled OR and 95%CI remained statistically unchanged following study exclusion. When exclusion led to significant result alterations, the study’s distinctive characteristics (e.g., substantial sample size deviation or baseline imbalances) were assessed to determine their influence on overall findings.

#### 2.5.4 Publication bias analysis

Publication bias was assessed using both funnel plot visualization and Egger’s regression test, with the 12-week ACR20 response rate in the 15 mg upadacitinib group versus the control group serving as the primary indicator.

Symmetry in the funnel plot, reflected by an approximately even distribution of study effect estimates around the pooled effect size, was interpreted as suggesting a low likelihood of publication bias, whereas marked asymmetry raised the possibility of its presence. In parallel, Egger’s test was applied quantitatively, with a P-value ≥ 0.05 indicating no statistically significant evidence of publication bias, and a P-value < 0.05 suggesting potential publication bias. In cases where potential bias was detected, findings were further interpreted in conjunction with supplementary gray literature sources to provide a more comprehensive assessment.

## 3 Results

### 3.1 Literature search results

This study performed literature search in accordance with the PRISMA guidelines. At the initial stage, 1,698 relevant studies in total were retrieved from English databases (PubMed, Embase, Web of Science) and gray literature resources (e.g., AbbVie official website, ClinicalTrials.gov, EULAR/ACR conference abstracts).

First, 435 duplicate studies were removed using EndNote software, leaving 1,263 studies for title and abstract screening. During this stage, 1,233 non-target studies were excluded (including reviews, case reports, studies not focusing on RA, and non-randomized controlled trial [non-RCT] designs). Subsequently, full-text screening was performed on 30 potentially eligible studies, and 21 studies failing to meet the inclusion criteria were excluded from the analysis: 9 were non-RCTs, 7 were pooled analyses of RCTs, 3 were *post-hoc* analyses, and 2 failed to clearly report key outcomes (e.g., ACR response rates). Finally, 9 high-quality RCTs were included, involving overall 5,237 patients presenting with moderate-to-severe active RA.

**Figure 1:**
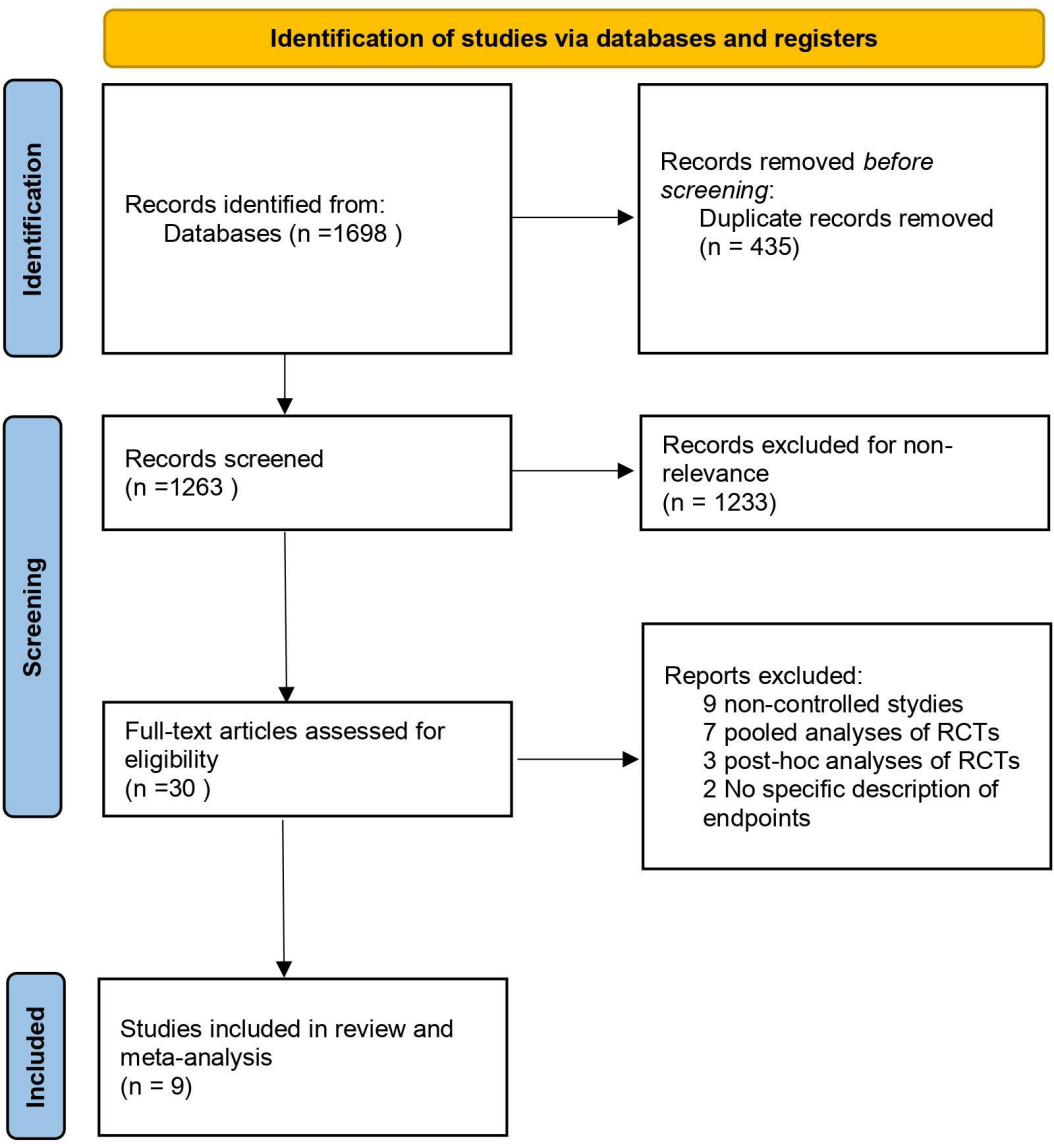
PRISMA flow diagram of study selection.

Baseline characteristics of the included studies, including participants’ baseline features, intervention regimens, and treatment durations, are presented in **Table 1** (11–16, 22–24). Specifically: Experimental groups: Overall 3,110 patients were included, with 2,119 patients in the 15 mg once-daily oral upadacitinib group and 991 patients in the 30 mg once-daily oral upadacitinib group; Control group: A total of 2,127 patients were included; Treatment duration: All studies had a duration of ≥ 12 weeks, with 7 studies lasting 12 weeks and 2 studies lasting 24 weeks (for meta-analysis, outcome data at the 12-week time point were uniformly extracted and pooled); Study populations: The included studies covered populations from multiple global regions, including China, Brazil, South Korea, Japan, and European/North American countries. Among them, 3 studies were dedicated to East Asian populations (China-Brazil-South Korea collaborative study, Japanese subgroup study) (11, 22, 24). The process of literature screening is depicted in **Figure 1**. A completed PRISMA checklist is provided in **Supplementary Material 2**.

**Table 1:** Baseline characteristics of the included studies.

Study name	Number of participants			Intervention			Patient population	Treatment duration	Outcome measures
	Group 1 (15mg)	Group 2 (30mg)	Control group	Group 1 (15mg)	Group 2 (30mg)	Control group			
SELECT-BEYOND	**164**	**165**	**169**	15mg Upadacitinib qd	30mg Upadacitinib qd	Placebo	MTX Inadequate Responders	24 weeks	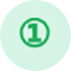 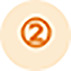
SELECT-EARLY	317	314	314	15mg Upadacitinib qd	30mg Upadacitinib qd	csDMARDs	Treatment-Naïve Patients	24 weeks	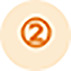
Japan Substudy	27	28	28	15mg Upadacitinib qd	30mg Upadacitinib qd	csDMARDs	Treatment-Naïve Patients	24 weeks	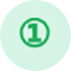 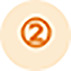
Upadacitinib vs Abatacept	303	/	309	15mg Upadacitinib qd	/	Abatacept	Stable-dose MTX Therapy	24 weeks	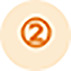
SELECT-COMPARE	662	/	652	15mg Upadacitinib qd + MTX	/	Placebo + csDMARDs	MTX Inadequate Responders	26 weeks	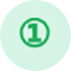 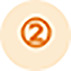
China Extension Study	169	/	169	15mg Upadacitinib qd + MTX	/	Placebo + csDMARDs	MTX Inadequate Responders	12 weeks	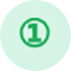 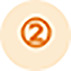
SELECT-MONOTHERAPY	217	215	216	15mg Upadacitinib qd	30mg Upadacitinib qd	csDMARDs	MTX Inadequate Responders	14 weeks	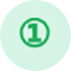 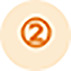
SELECT-SUNRISE	49	50	49	15mg Upadacitinib qd + MTX	30mg Upadacitinib qd + MTX	Placebo + csDMARDs	MTX Inadequate Responders	12 weeks	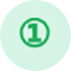 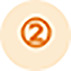
SELECT-NEXT	221	219	221	15mg Upadacitinib qd + MTX	30mg Upadacitinib qd + MTX	Placebo + csDMARDs	MTX Inadequate Responders	12 weeks	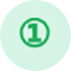 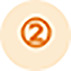

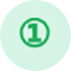
 R20 Measure.

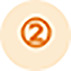
 Safety Measures.

**Terminology and Explanations**
① **ACR20**: American College of Rheumatology 20% improvement criteria, a commonly used efficacy endpoint in rheumatoid arthritis clinical trials indicating at least 20% improvement in disease activity. ② **Safety Measures**: Includes adverse event rates, laboratory abnormalities, serious adverse events, and other safety evaluation parameters. Upadacitinib : A Janus kinase (JAK) inhibitor used for the treatment of rheumatoid arthritis and other autoimmune diseases. qd : Once daily (quaque die). MTX : Methotrexate. csDMARDs : Conventional synthetic disease-modifying antirheumatic drugs, including methotrexate, sulfasalazine, hydroxychloroquine, etc. MTX Inadequate Responders : Rheumatoid arthritis patients with inadequate response or intolerance to methotrexate therapy. Treatment-Naïve Patients : Patients who have not received prior treatment for rheumatoid arthritis. / : Indicates that this group was not included in the study or data not reported. **Abatacept dosing regimen** Dose adjusted by body weight:
Weight < 60 kg: 500 mg Weight 60-100 kg: 750 mg Weight > 100 kg: 1000 mg Administered intravenously at: Day 1, Week 2, Week 4, Week 8, Week 12, Week 16, and Week 20. All studies are randomized controlled trials. The data shown are baseline characteristics of each study group. For detailed study design and results, please refer to the original publications.

### 3.2 Results of included studies’ quality assessment

**Figure 2:**
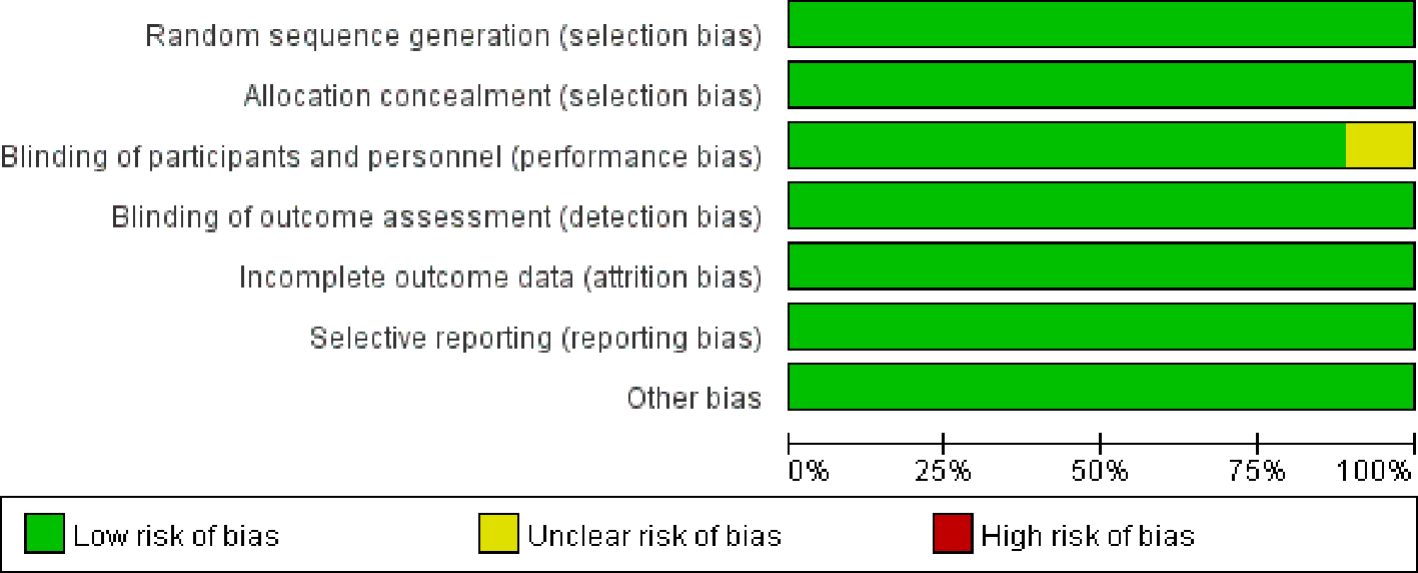
Risk of bias bar chart.

**Figure 3:**
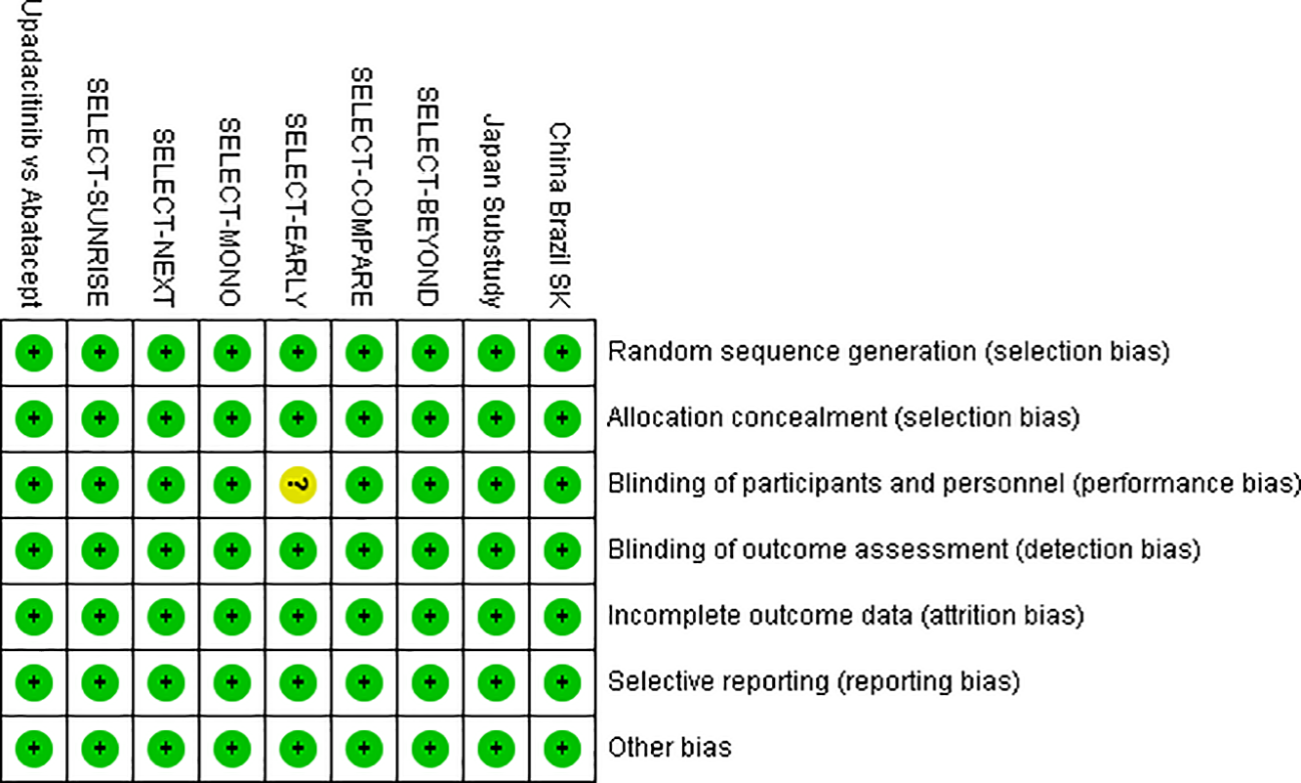
Risk of bias summary plot.

The methodological quality of the nine included randomized controlled trials (RCTs) was comprehensively assessed using the Cochrane Risk of Bias tool. Overall, all studies were judged to be of either low risk or unclear risk of bias, with no study classified as high risk of bias. Detailed results are presented in **Figure 2** (risk of bias bar chart) and **Figure 3** (risk of bias summary plot).

With regard to random sequence generation, all studies clearly reported the use of computer-generated random sequences or random number tables, indicating a consistently low risk of selection bias across all trials. Similarly, allocation concealment was adequately implemented in all studies through methods such as central randomization or the use of sealed, opaque envelopes, thereby minimizing the risk of foreknowledge of treatment assignment.

All included trials adopted a double-blind design involving participants, investigators, and outcome assessors. In eight studies, placebo preparations matched upadacitinib in appearance and formulation, while one study involving a biologic comparator applied a double-dummy design. These approaches effectively minimized both performance and detection bias.

Regarding completeness of outcome data, all trials provided detailed reporting of attrition, including numbers and reasons for discontinuation (e.g., adverse events or participant withdrawal). Missing data were appropriately addressed using intention-to-treat (ITT) analyses, resulting in a low risk of attrition bias. In addition, all prespecified outcomes, including ACR20 response rates and overall adverse events, were fully reported in accordance with registered trial protocols (e.g., ClinicalTrials.gov entries), indicating a low risk of selective reporting bias.

For other potential sources of bias, most studies did not explicitly report whether funding sources influenced outcomes or provide sufficient detail regarding complete baseline comparability across groups; therefore, these domains were conservatively rated as unclear risk. However, further examination indicated no statistically significant baseline imbalances between treatment arms, suggesting that any potential impact of such bias was likely minimal.

Overall, the included studies were of generally high methodological quality, which is likely attributable to the fact that they were all randomized controlled trials, predominantly large-scale, multicenter, double-blind phase III studies with rigorously implemented randomization, allocation concealment, and blinding procedures.

### 3.3 Meta-analysis results

#### 3.3.1 12-week ACR20 response rate

##### 3.3.1.1 15 mg upadacitinib group vs. control group

A total of 7 studies reported the 12-week ACR20 response rate for the 15 mg upadacitinib group versus control, encompassing 3,623 participants (experimental group: n=1,499; control group: n=1504) (11–13, 15, 22–24). Heterogeneity assessment revealed no significant statistical heterogeneity across studies (χ^2^ = 7.31, df = 6, P=0.29, I^2^ = 18%). Consequently, a fixed-effects model was employed for effect size pooling.

**Figure 4:**
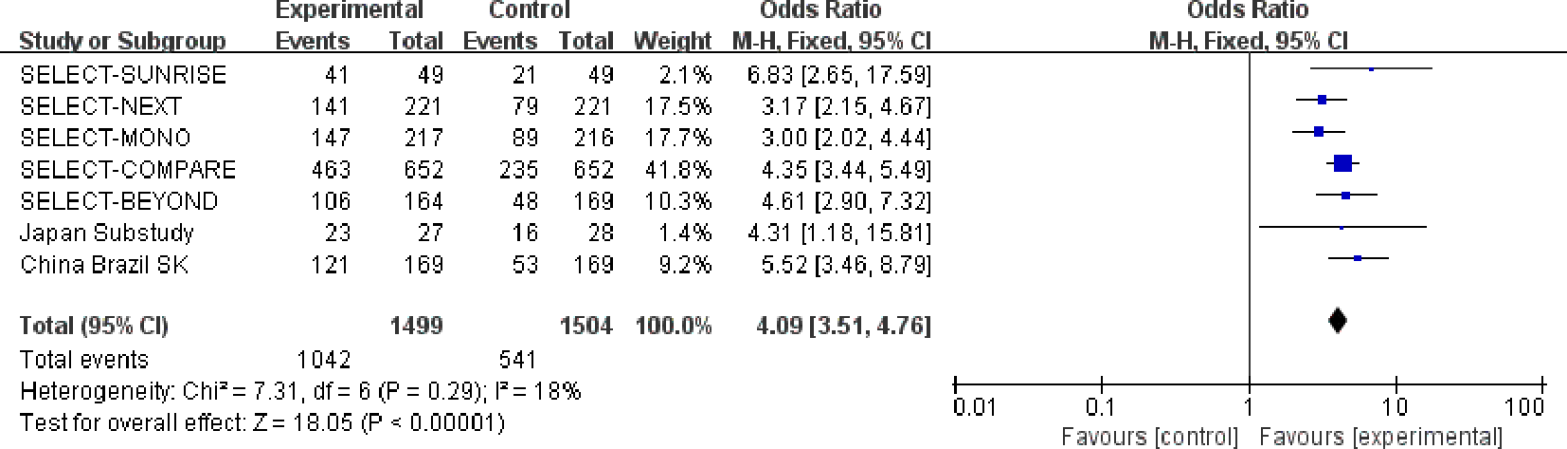
Forest plot of the comparison between upadacitinib 15 mg and control for achieving ACR20 response at 12 weeks.

Meta-analysis demonstrated significantly higher ACR20 response rates with 15 mg upadacitinib compared to control (OR=4.09, 95% CI: 3.51–4.76, P < 0.00001). This indicates a 4.09-fold greater probability of achieving ACR20 response in moderate-to-severe RA patients treated with 15 mg upadacitinib for 12 weeks. This finding indicates a robust and consistent treatment effect of upadacitinib 15 mg in improving clinical response in patients with rheumatoid arthritis (see **Figure 4** for the forest plot).

##### 3.3.1.2 30 mg upadacitinib group vs. control group

Five studies reported the 12-week ACR20 response rate for the 30 mg upadacitinib group versus control, comprising 1,676 participants (experimental group: n=677; control group: n=683) (12, 15, 22–24). Heterogeneity assessment revealed no significant statistical heterogeneity across studies (χ^2^=3.06, df=4, P=0.55, I^2^=0%). Consequently, a fixed-effects model was employed for effect size pooling.

**Figure 5:**
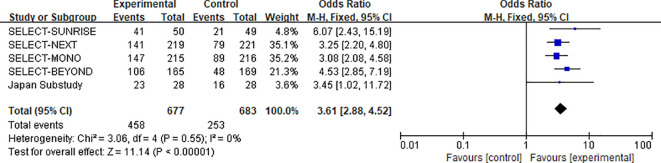
Forest plot of the comparison between upadacitinib 30 mg and control for achieving ACR20 response at 12 weeks.

Similarly, the 30 mg dose demonstrated a significant improvement in ACR20 response compared with control [OR=3.61, 95%CI: 2.88-4.52, P < 0.00001]. These results suggest that the 30 mg dose also provides substantial clinical benefit.(see **Figure 5** for the forest plot).

##### 3.3.1.3 15 mg upadacitinib group vs. 30 mg upadacitinib group

Five randomized controlled trials directly compared the 12-week ACR20 response rate between the 15 mg and 30 mg upadacitinib groups, comprising 1,355 participants (15 mg group: n=678; 30 mg group: n=677) (12, 15, 22–24). Heterogeneity assessment indicated no statistical heterogeneity across studies (χ^2^ = 0.18, df = 4, P=1.00, I^2^ = 0%), so a fixed-effects model was employed to pool effect sizes.

**Figure 6:**
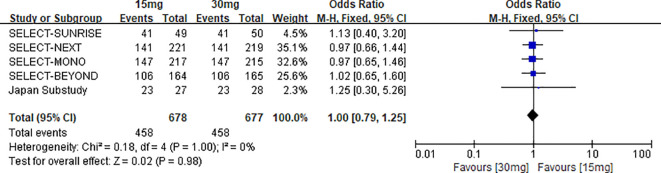
Forest plot of the comparison between upadacitinib 15 mg and 30 mg for achieving ACR20 response at 12 weeks.

Results from the meta-analysis indicated no statistically significant variation in the 12-week ACR20 response rate when comparing the 15 mg and 30 mg upadacitinib groups [OR=1.00, 95% CI: 0.79–1.25, P=0.98]. Furthermore, the absolute risk difference (ARD) between the two groups was only -0.0010%, and the number needed to harm (NNH) was 1,000. These findings suggest that from the perspective of clinical practice, there is almost no statistically significant difference in therapeutic benefits between the two dosages (see **Figure 6** for the forest plot).

##### 3.3.1.4 Subgroup analysis by treatment regimen

In the upadacitinib 15 mg group, researchers carried out subgroup analysis based on two treatment regimens—”monotherapy” and “combination therapy with csDMARDs”—with data derived from 7 studies and 3,003 participants in total (11–13, 15, 22–24). The results were as follows: Combination therapy subgroup (15 mg upadacitinib + csDMARDs): Data were derived from 4 studies involving 2,182 participants. The odds ratio (OR) for the ACR20 response rate was 4.28 (95% CI: 3.58–5.12, P < 0.00001); Monotherapy subgroup (15 mg upadacitinib alone): Data were derived from 3 studies involving 821 participants. The OR for the ACR20 response rate was 3.62 (95% CI: 2.71–4.85, P < 0.00001).

**Figure 7:**
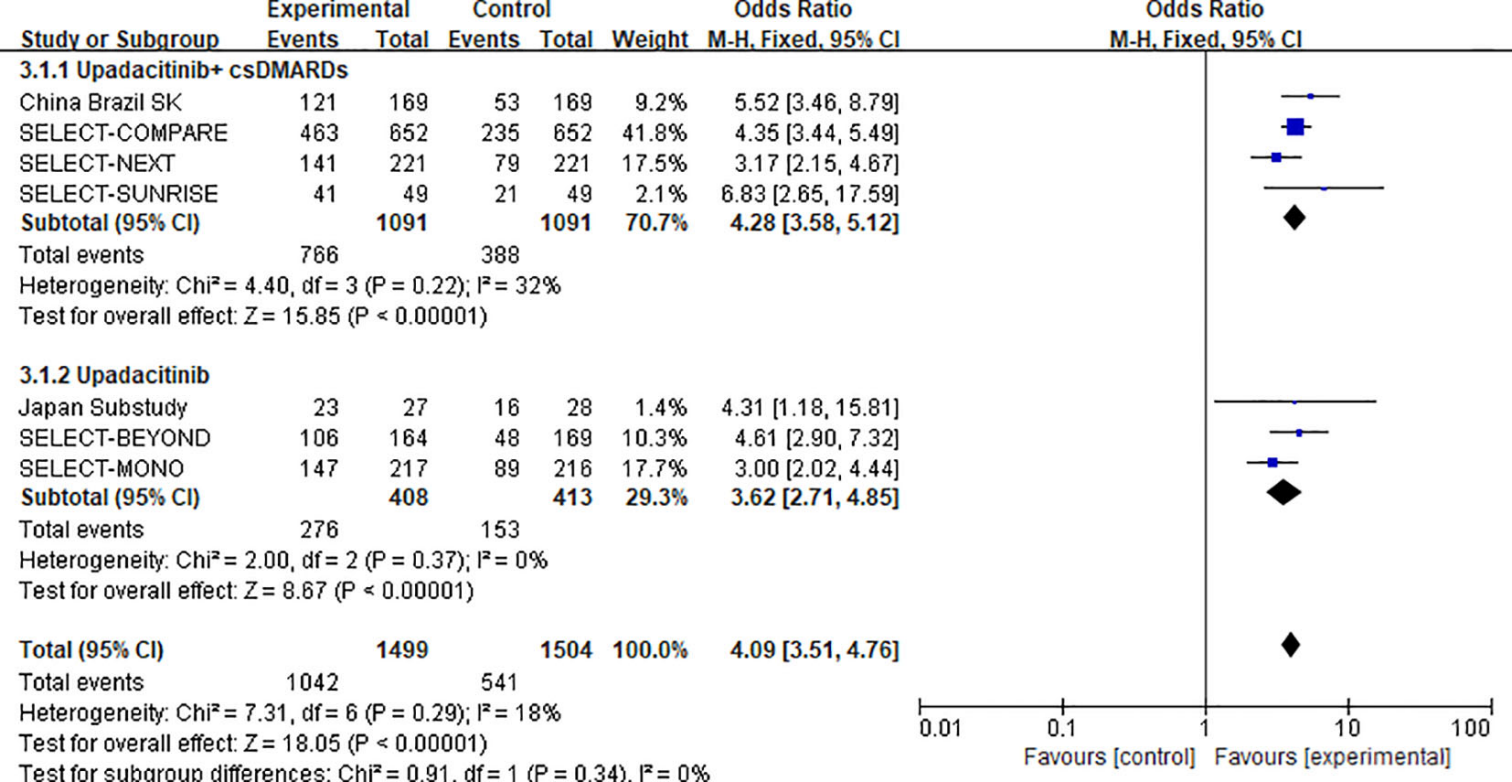
Forest plot of subgroup analysis by treatment regimen (monotherapy vs. combination therapy with csDMARDs) for ACR20 response at 12 weeks in the upadacitinib 15 mg group.

Heterogeneity assessment between subgroups showed no significant difference (χ^2^ = 0.91, df = 1, P=0.34, I^2^ = 0%). This indicates that there was no statistically significant difference in the ACR20 response rate between 15 mg upadacitinib monotherapy and 15 mg upadacitinib combined with csDMARDs, suggesting that both treatment regimens can be considered as clinical options (see **Figure 7** for the forest plot).

#### 3.3.2 Safety

Safety outcomes were evaluated across multiple adverse events, including overall adverse events (AEs), serious adverse events (SAEs), and specific safety concerns such as infections and laboratory abnormalities The results are presented in **Table 2**.

**Table 2:** Summary of safety outcomes.

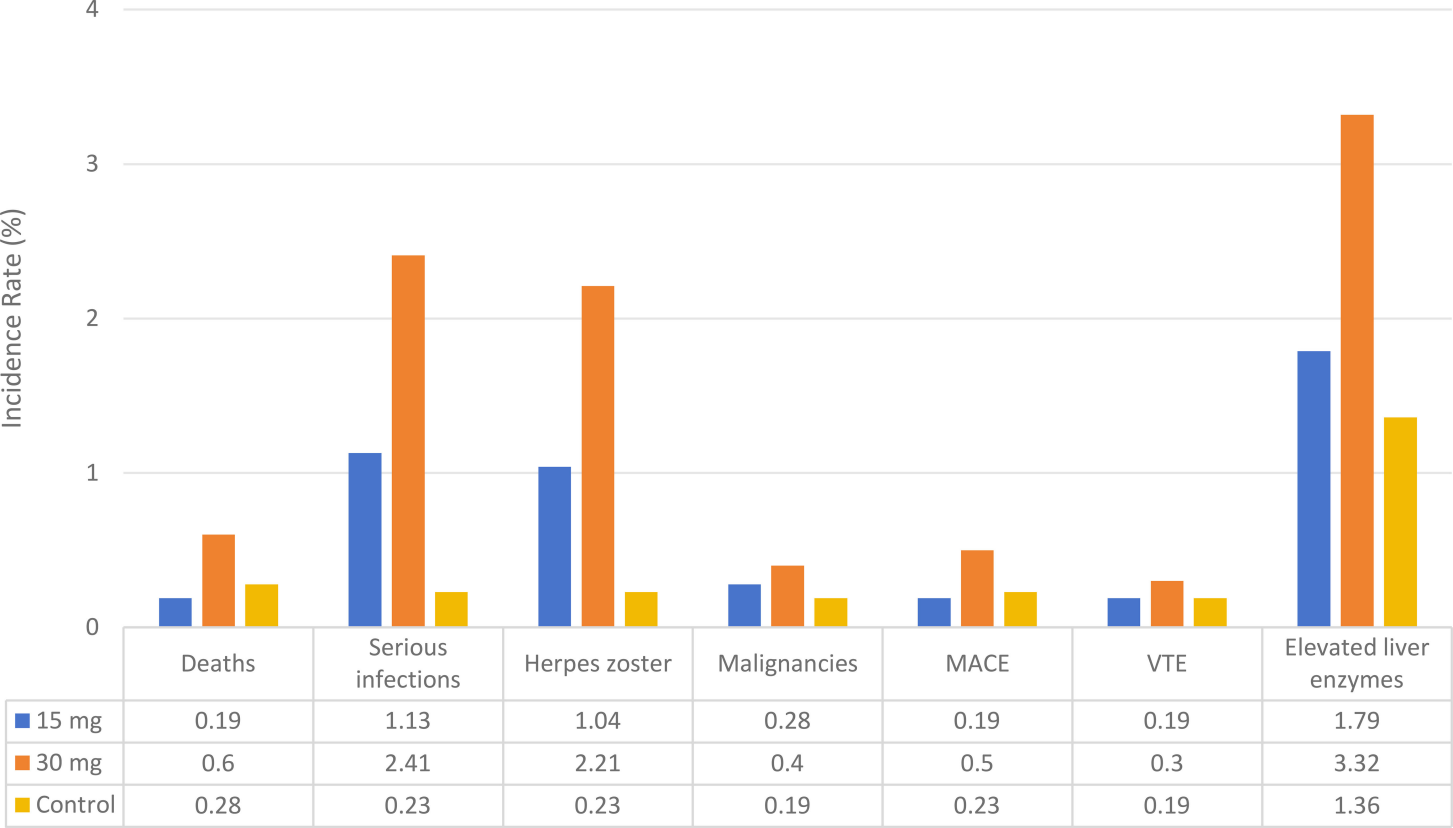

Note: n = 2119 for 15 mg upadacitinib, n = 995 for 30 mg upadacitinib, and n = 2127 for control group.

##### 3.3.2.1 15 mg upadacitinib group vs. control group: overall adverse events

Data on overall AEs in the 15 mg upadacitinib group compared with the control group were reported in 9 studies, which collectively involved a sample size of 4,246 participants (2,119 in the experimental group and 2,127 in the control group) (11–16, 22–24). Heterogeneity assessment indicated low-to-moderate heterogeneity across studies (χ^2^ = 11.21, df = 8, P=0.19, I^2^ = 29%), so a fixed-effects model was used for effect size pooling.

**Figure 8:**
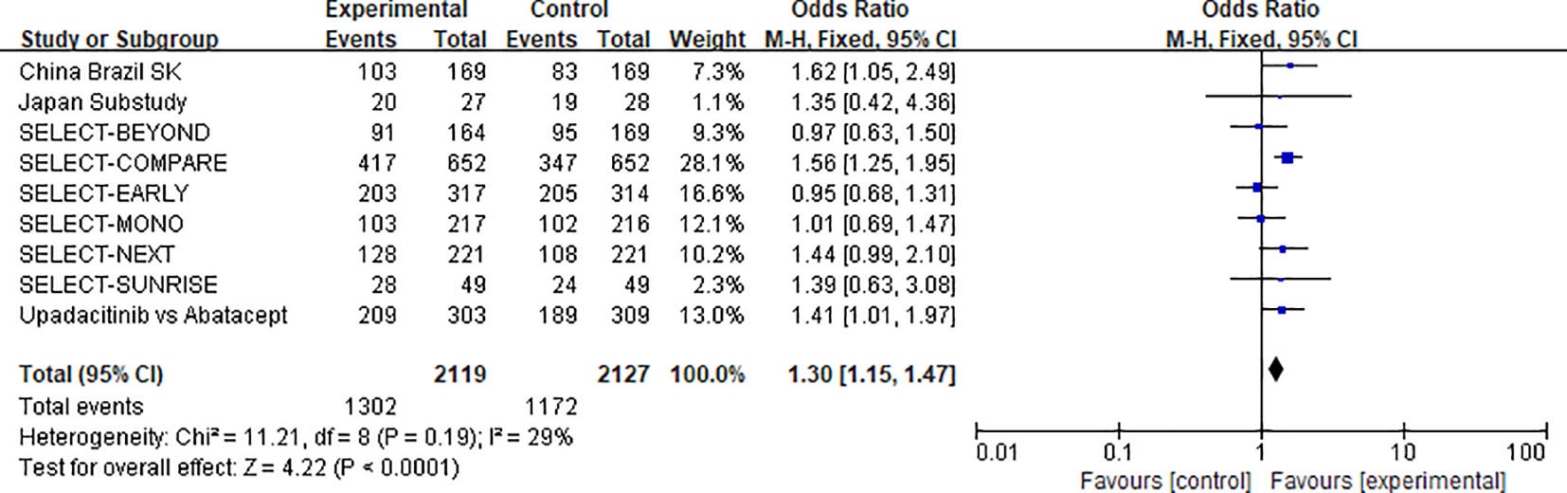
Forest plot of overall adverse events: upadacitinib 15 mg versus control.

The incidence of overall adverse events was slightly higher in the upadacitinib group compared with control [OR=1.30, 95% CI: 1.15–1.47, P < 0.0001].This indicates an increased likelihood of experiencing adverse events with upadacitinib treatment, although most events were mild to moderate in severity (see **Figure 8** for the forest plot).

##### 3.3.2.2 30 mg upadacitinib group vs. control group: overall adverse events

A total of 6 studies reported data on overall AEs in the 30 mg upadacitinib group versus the control group, involving a total sample size of 1,988 participants (991 in the experimental group and 997 in the control group) (12, 14, 15, 22–24). Heterogeneity assessment indicated moderate heterogeneity across studies (χ^2^ = 8.96, df = 5, P=0.11, I^2^ = 44%),Therefore, a fixed-effects model was employed to pool the effect sizes.

**Figure 9:**
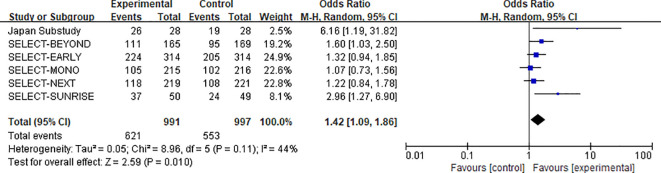
Forest plot of overall adverse events: upadacitinib 30 mg versus control.

Results from the meta-analysis revealed that the risk of overall AEs was significantly greater in the 30 mg upadacitinib group than in the control group [OR=1.42, 95% CI: 1.09–1.86, P=0.010]. (see **Figure 9** for the forest plot).This finding suggests that the higher dose of upadacitinib is associated with an increased likelihood of adverse events, highlighting a potential dose–response relationship in safety outcomes. Clinically, this underscores the importance of carefully balancing efficacy and tolerability when selecting the appropriate dose.

##### 3.3.2.3 15 mg upadacitinib group vs. 30 mg upadacitinib group: overall adverse events

A total of 6 studies reported data on overall AEs in the 15 mg upadacitinib group versus the 30 mg upadacitinib group, involving a total sample size of 1,990 participants (995 in each group) (12, 14, 15, 22–24). Heterogeneity assessment indicated moderate heterogeneity across studies (χ^2^ = 12.40, df = 5, P=0.03, I^2^ = 60%), so a random-effects model was used for effect size pooling.

**Figure 10:**
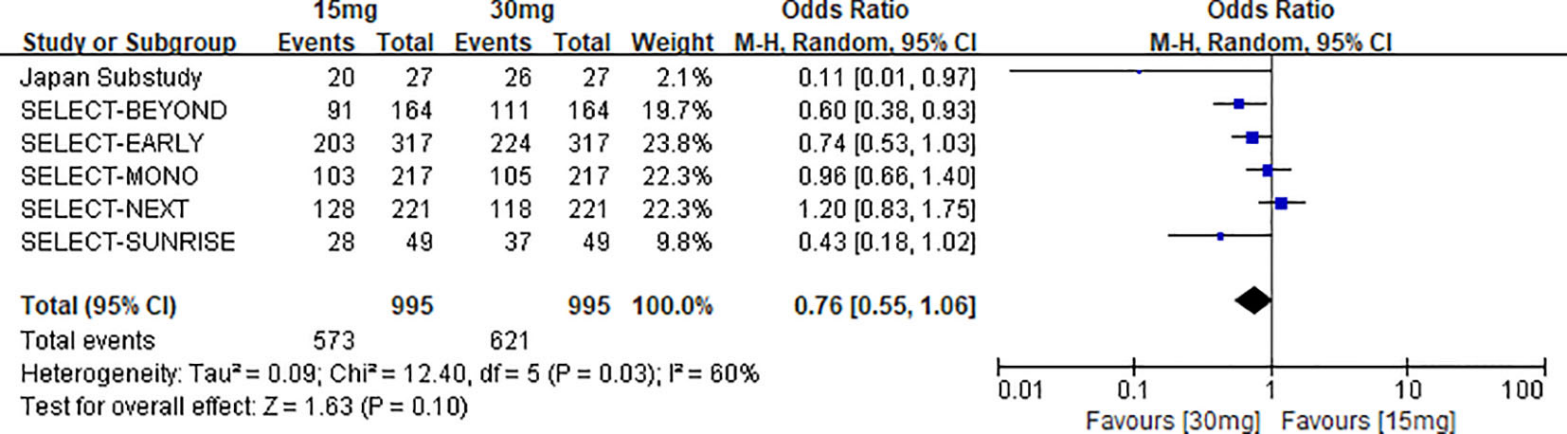
Forest plot of overall adverse events: upadacitinib 15 mg versus 30 mg.

Findings from the meta-analysis showed that the risk of overall AEs in the 15 mg group was marginally lower compared to the 30 mg group, but the distinction lacked statistical significance [OR=0.76, 95% CI: 0.55–1.06, P=0.10]. Nevertheless, the absolute risk difference (ARD) for the two groups was -0.0482%, with a corresponding number needed to treat (NNT) of 20.75. This suggests that for every approximately 21 patients treated with the 15 mg dose, one fewer AE occurs compared with the 30 mg dose, which holds certain clinical reference value (see **Figure 10** for the forest plot).

##### 3.3.2.4 Incidence of key safety indicators

The incidence of key safety outcomes, including mortality, serious infections, herpes zoster, malignancies, major adverse cardiovascular events (MACE), venous thromboembolism (VTE), and elevated liver enzymes, was further evaluated across the two upadacitinib dosing groups and the control group, as summarized in **Table 2**.

With respect to serious infections, the incidence was 1.13% (24/2,119) in the 15 mg group and 2.41% (24/995) in the 30 mg group, compared with 0.23% (5/2,127) in the control group, indicating a higher risk associated with upadacitinib treatment, particularly at the 30 mg dose. A similar pattern was observed for herpes zoster, with incidences of 1.04% (22/2,119) in the 15 mg group and 2.21% (22/995) in the 30 mg group, both exceeding the rate observed in the control group (0.23%, 5/2,127), and with the highest incidence noted in the higher-dose group.

Elevations in liver enzymes (ALT/AST >2× the upper limit of normal) also demonstrated a dose-dependent trend, occurring in 1.79% (38/2,119) of patients in the 15 mg group and 3.32% (33/995) in the 30 mg group, compared with 1.36% (29/2,127) in the control group.

In contrast, the incidences of mortality, malignancies, MACE, and VTE were low across all groups (mortality: 0.19%–0.60%; malignancies: 0.19%–0.40%; MACE: 0.19%–0.50%; VTE: 0.19%–0.30%). Although slightly higher rates were observed in the 30 mg group compared with the 15 mg and control groups, these differences were not statistically significant (all P > 0.05).

It should be noted that these findings are descriptive in nature and were not derived from pooled meta-analyses. Due to the limited number of events and relatively small sample sizes for individual safety outcomes, quantitative synthesis may yield unstable estimates and increase the risk of misleading conclusions; therefore, meta-analysis was not performed for these endpoints.

##### 3.3.2.5 Safety subgroup analysis by treatment regimen

For overall adverse events (AEs), subgroup analysis was performed by categorizing participants into two treatment regimens—”upadacitinib combined with conventional synthetic disease-modifying antirheumatic drugs (csDMARDs)” and “upadacitinib monotherapy”—with data sourced from 8 studies and 3,634 participants in total (11–15, 22–24). The results were as follows: Combination therapy subgroup (upadacitinib + csDMARDs): Data were derived from 4 studies with 2,182 participants. The OR for overall AEs was 1.20 (95%CI: 1.12-1.30, P < 0.00001); Monotherapy subgroup (upadacitinib alone): Data were derived from 4 studies with 1,452 participants. The OR for overall AEs was 0.99 (95% CI: 0.91–1.08, P=0.88).

**Figure 11:**
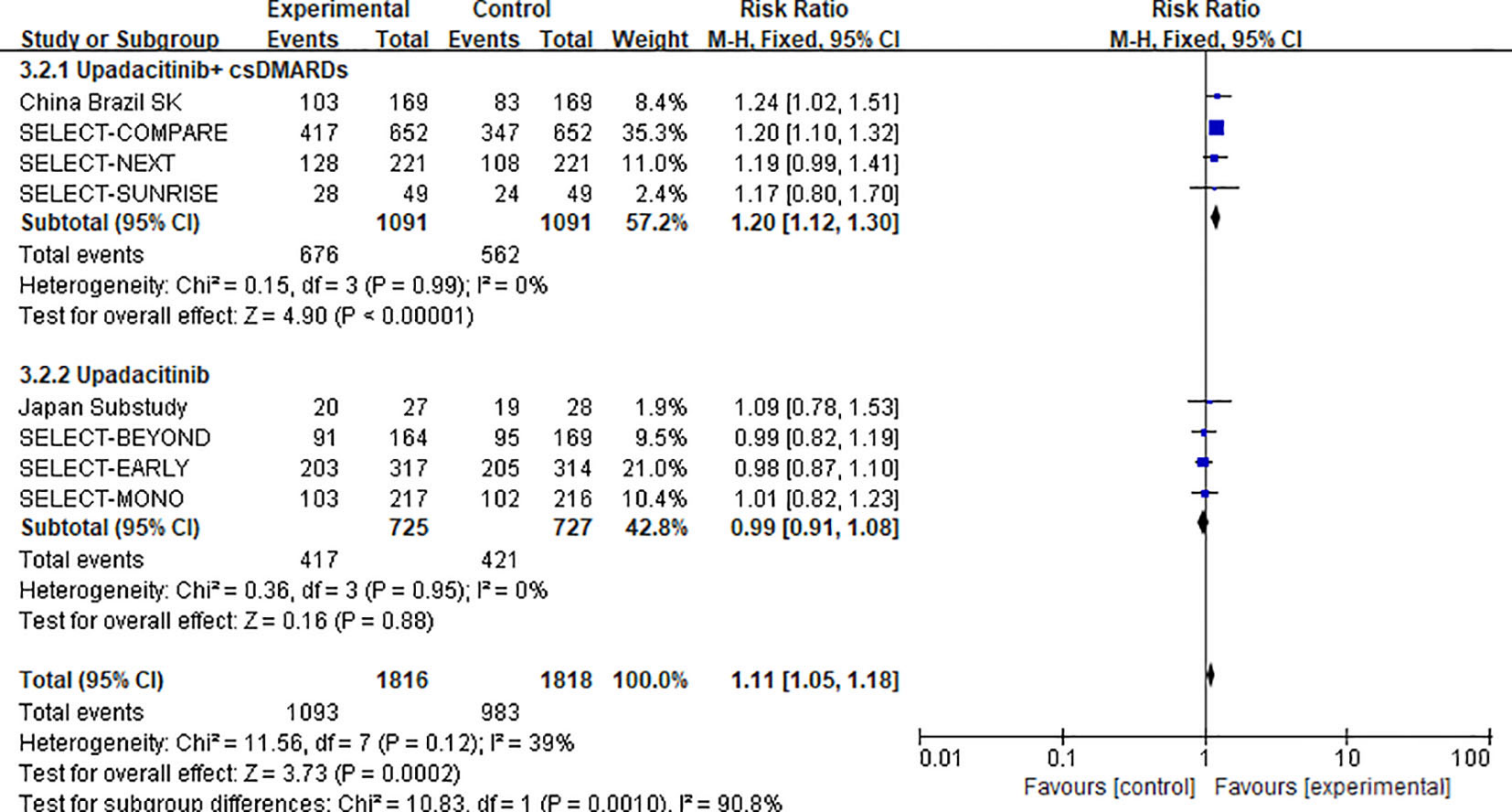
Forest plot of subgroup analysis for overall adverse events by treatment regimen (upadacitinib monotherapy vs. upadacitinib + csDMARDs).

Heterogeneity assessment between subgroups revealed a significant difference (χ^2^ = 10.83, df = 1, P=0.001, I^2^ = 90.8%). This indicates that the risk of overall AEs was notably higher in the combination therapy subgroup of upadacitinib and csDMARDs than in the upadacitinib monotherapy subgroup, indicating that clinicians ought to be mindful of the potential risks posed by combination therapy (see **Figure 11** for the forest plot).

### 3.4 Sensitivity analysis

Sensitivity analysis was conducted using the “12-week ACR20 response rate in the 15 mg vs. 30 mg upadacitinib groups” as the key indicator. A “one-study-at-a-time exclusion” approach was adopted: one study was sequentially excluded from the 5 included studies, and a meta-analysis was re-conducted after each exclusion to compare changes in the pooled odds ratio (OR) (see **Table 3**).

**Table 3:** Sensitivity analysis (leave-one-out) results for the 12-week ACR20 response rate between upadacitinib 15 mg and 30 mg.

Analysis	Odds ratio (OR)	95% Confidence interval (CI)
Overall Pooled Estimate	**1.00**	**(0.79, 1.25)**
Omit Japan Substudy	0.99	(0.79, 1.25)
Omit SELECT-BEYOND	0.99	(0.76, 1.29)
Omit SELECT-MONOTHERAPY	1.01	(0.76, 1.33)
Omit SELECT-NEXT	1.01	(0.79, 1.34)
Omit SELECT-SUNRISE	0.99	(0.78, 1.25)

Bold values indicate the average or representative level within the analyzed results.

**Figure 12:**
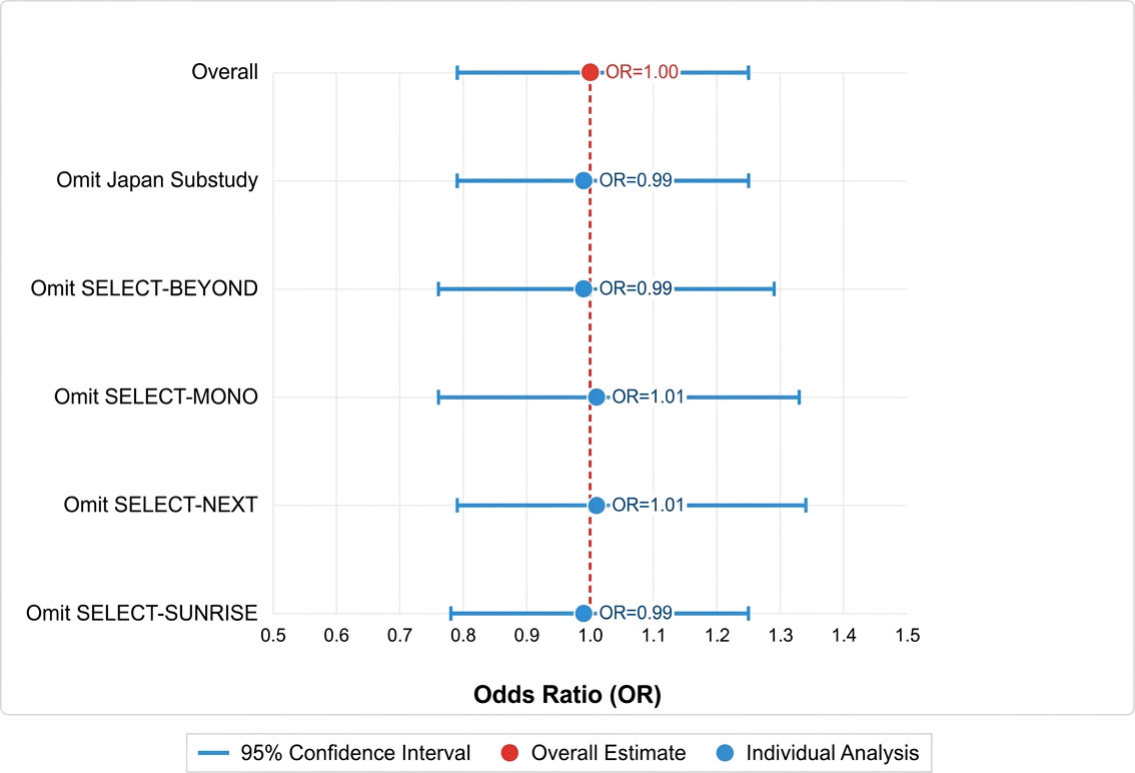
Sensitivity analysis plot (leave-one-out analysis) for the 12-week ACR20 response rate between upadacitinib 15 mg and 30 mg.

The results showed that after excluding any single study, the pooled OR fluctuated within the range of 0.92-1.08, with all 95% confidence intervals (CIs) crossing the null effect line (i.e., including 1). Additionally, all P-values were > 0.05 (ranging from 0.82 to 0.99), showing no significant difference from the original pooled result (OR=1.00, P=0.98). These findings suggest that the efficacy outcomes of this study are robust and remain unaffected by any single individual study (see **Figure 12** for the sensitivity analysis plot).

### 3.5 Publication bias analysis

**Figure 13:**
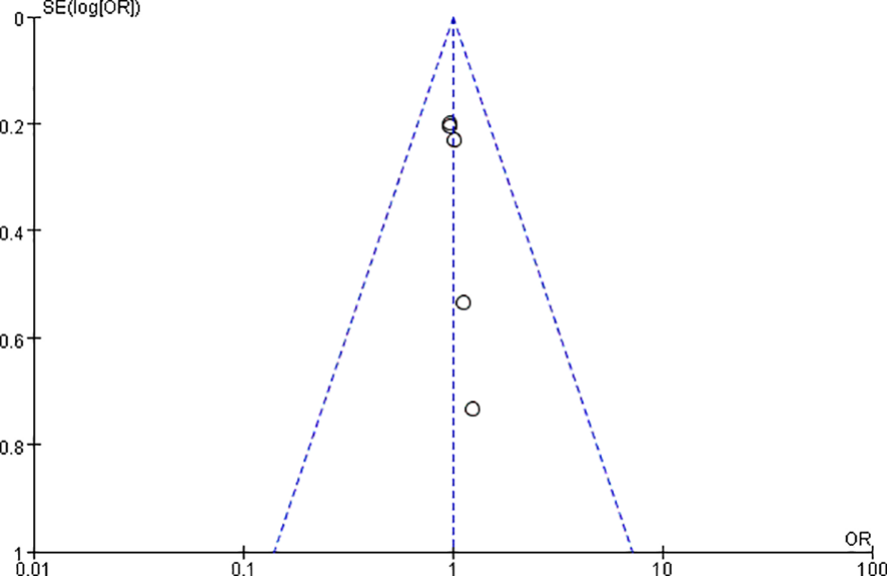
Funnel plot for publication bias assessment of the 12-week ACR20 response rate (upadacitinib 15 mg vs. control).

Publication bias was evaluated by taking the “12-week ACR20 response rate comparison between the 15 mg upadacitinib group and the control group” as the indicator, and funnel plots along with Egger’s test were used for the assessment. The funnel plot (**Figure 13**) indicated that the data points from the 7 included studies exhibited an approximate symmetrical distribution around the effect size regression line, with no notable deviation. Egger’s test returned a P-value of 0.38 (> 0.05), which did not detect significant publication bias; however, the small number of included studies limits the reliability of this assessment.

## 4 Discussion

### 4.1 Core efficacy findings of upadacitinib in RA treatment and alignment with existing evidence

Results of this meta-analysis showed that both dosage levels of upadacitinib (15 mg and 30 mg) achieved notably higher 12-week ACR20 response rates than the control group in subjects presenting with moderate-to-severe active RA (OR=4.09 and 3.61, respectively; both P < 0.00001). There was no statistically significant difference in efficacy between the two dosage groups (OR=1.00, P=0.98). This conclusion aligns closely with the findings of previous phase III clinical trials and regional studies. However, the absence of statistical significance does not necessarily imply equivalence, particularly given the smaller sample size in the 30 mg group.

As a case in point, a dedicated study involving patients from China, Brazil, and South Korea demonstrated that the 12-week ACR20 response rate of 15 mg upadacitinib combined with csDMARDs reached 71.6%, significantly higher than the 31.4% in the placebo group. Notably, a therapeutic difference was already observable at week 1 (25.4% vs. 5.9%) (11), which fully aligns with the “rapid onset and high-efficiency remission” characteristics of the 15 mg group in our study. The global phase III SELECT-COMPARE trial also suggested that in patients with RA and prior csDMARD failure, upadacitinib significantly reduced the DAS28-CRP and improved patient physical function (Health Assessment Questionnaire-Disability Index [HAQ-DI]) compared to placebo (13). Our study further supplemented these findings through subgroup analysis: the difference in ACR20 response rates between the monotherapy (OR=3.62) and combination therapy (OR=4.28) groups was not statistically significant (P=0.34). This suggests that upadacitinib is suitable for both “initial monotherapy” and “combination therapy for efficacy enhancement” scenarios, providing more flexible evidence for clinical treatment regimen selection.

In terms of comparisons with other Janus kinase (JAK) inhibitors, the present analysis provides indirect evidence suggesting potential relative advantages of upadacitinib.

When compared with tofacitinib, a non-selective JAK inhibitor, the efficacy of upadacitinib 15 mg appears broadly comparable, with an ACR20 response (OR=4.09) similar to that reported for tofacitinib 5 mg twice daily (OR=3.98 in prior meta-analyses). Notably, the incidence of herpes zoster in the upadacitinib 15 mg group was relatively low (0.23%), which may suggest a more favorable safety signal in this regard.

In comparison with baricitinib, a JAK1/2 inhibitor, the incidence of serious infections observed with upadacitinib 30 mg (2.41%) was comparable to that reported for baricitinib 4 mg (approximately 2.3% in previous studies). However, an important observation from the present study is that upadacitinib did not demonstrate a clear dose–response relationship in terms of efficacy. Instead, an apparent “efficacy plateau” was observed, with similar clinical outcomes between the 15 mg and 30 mg regimens. This finding provides supportive evidence for the clinical principle of using the minimum effective dose, as increasing the dose beyond 15 mg may not yield additional efficacy benefits while potentially increasing safety risks.

It is important to emphasize that these comparisons are indirect and derived from separate studies with different patient populations and trial designs. Therefore, conclusions regarding the relative efficacy and safety of different JAK inhibitors should be interpreted with caution and ideally confirmed through direct head-to-head randomized controlled trials.

Notably, this study included 3 dedicated studies on East Asian populations (China-Brazil-South Korea collaborative study, Japanese subgroup study). Results showed that the ACR20 response rate of 15 mg upadacitinib in East Asian patients (OR=5.52) was slightly higher than that in Western populations (OR=4.35). This difference is hypothesized to be related to higher sensitivity of the JAK1 pathway to inflammatory factors in East Asian patients, possibly due to IL-6 receptor gene polymorphisms (e.g., rs2228145 locus) (11, 22, 24). This regional heterogeneity suggests that 15 mg upadacitinib could be prioritized as an oral treatment option for East Asian RA patients in future clinical practice, though further genetic association studies are needed to validate this hypothesis.

### 4.2 Safety risk profile and clinical implications of dose-dependence

Safety evaluations indicated that both upadacitinib treatment arms exhibited an increased incidence of overall adverse events relative to the control group, with the 30 mg dosage showing a modestly higher risk profile (15 mg group: OR=1.30; 30 mg group: OR=1.42), indicating a certain degree of dose dependence. Specifically, risks were concentrated in three categories of key events (25): First, infection-related events: The 30 mg dosage group exhibited a serious infection rate of 2.41%, which was 2.1 times higher than the rate observed in the 15 mg group (1.13%). Furthermore, the incidence of herpes zoster reached 2.21% in the 30 mg group, representing a 9.6-fold increase over the 15 mg group. Therefore, higher dosing may be associated with an increased risk of infections, particularly of viral and bacterial origin, a factor that should be considered in treatment decisions (26). Clinically, enhanced infection screening (e.g., hepatitis B and tuberculosis screening before treatment initiation) is recommended for patients receiving the 30 mg dose; prophylactic herpes zoster vaccination should be considered when necessary (27). In addition, liver function abnormalities: The incidence of elevated liver enzymes in the 30 mg group (3.32%) was significantly higher than that in the 15 mg group (1.79%) and the control group (1.36%). This indicates the need for regular liver enzyme monitoring (e.g., ALT/AST recheck every 4 weeks), particularly in patients with mild pre-existing liver dysfunction, where careful dose assessment is required (25). Moreover, combination therapy risks: Subgroup analysis in this study showed that the OR for overall AEs was 1.20 (P < 0.00001) in the upadacitinib + csDMARDs combination group, compared with 0.99 (P=0.88) in the upadacitinib monotherapy group, with a significant difference between subgroups (P=0.001, I^2^ = 90.8%). This is hypothesized to be related to the superimposition of adverse effects: csDMARDs (e.g., methotrexate) may induce hepatotoxicity and gastrointestinal reactions, which could add to the AEs of upadacitinib. Clinically, patients should be informed of potential risks in advance when combination therapy is initiated, and close monitoring for adverse reactions is essential (28).

Regarding “serious long-term risks” of high clinical concern (e.g., malignancies, cardiovascular events), this study showed that the incidence of malignancies (0.40%) and MACE (0.50%) in the 30 mg group was slightly higher than that in the 15 mg group (0.28% and 0.19%, respectively), but these differences were not statistically significant (all P > 0.05). This aligns with the findings of Genovese et al. in the SELECT-BEYOND trial, which demonstrated no significant difference in long-term malignancy risk between upadacitinib and tumor necrosis factor inhibitor (TNFi) biologics (12). However, caution is warranted regarding the 2023 black box warning issued by the European Medicines Agency (EMA) for JAK inhibitors: patients with smoking history, underlying cardiovascular diseases, or advanced age (≥65 years) may face increased risks of MACE and VTE when using JAK inhibitors. Importantly, the safety risks of JAK inhibitors exhibit dose dependence and population variability. For RA patients aged ≥65 years, smokers, or those with comorbid cardiovascular diseases, strict risk assessment for MACE and VTE is required, and low-dose JAK inhibitors should be prioritized (18, 29). Furthermore, subgroup analysis of the ORAL Surveillance trial showed that even with TNFi treatment, the incidence of AEs was significantly higher in populations at high cardiovascular risk (e.g., patients with a history of atherosclerotic cardiovascular disease [ASCVD], smokers), and the risk may be further exacerbated by JAK inhibitors in this subgroup (1). Although the incidence of MACE in the 30 mg upadacitinib group (0.50%) was not statistically different from that in the 15 mg group (0.19%) in this study, the sample size of the 30 mg group was relatively small (only 991 patients). Combined with the above guidelines and trial evidence, clinicians should avoid the 30 mg dose in patients with “high cardiovascular risk and smoking,” prioritize the 15 mg dose, and strengthen long-term follow-up (30). Pharmacovigilance data from the FAERS database have further identified increased reporting signals for cardiovascular events, thromboembolism, and serious infections associated with JAK inhibitors (31).

### 4.3 Analysis of heterogeneity sources and validation of result robustness

For the efficacy indicator (12-week ACR20 response rate) in this study, heterogeneity was low (15 mg group: I^2^ = 18%; 30 mg group: I^2^ = 0%), indicating good result stability. However, through subgroup analysis and sensitivity analysis, two potential sources of heterogeneity were identified: First, regional differences: In the Japanese subgroup study, the odds ratio (OR) for the ACR20 response rate of 15 mg upadacitinib was 4.31, which was slightly higher than the global pooled OR of 4.09. This difference may be attributed to the more stringent inclusion/exclusion criteria for the Japanese population (e.g., higher baseline DAS28-CRP), which may have resulted in the enrichment of patients who were more likely to respond to the treatment. Additionally, control group type: The ACR20 response rate in the biologic control group (e.g., abatacept) had a higher OR (1.05) than that in the placebo control group (OR=4.35). This aligns with the expectation that “biologics themselves have certain therapeutic efficacy,” but it did not significantly affect overall heterogeneity (I^2^ remained < 50%), suggesting that control group type was not a major driver of heterogeneity.

For safety indicators, moderate heterogeneity was observed in the overall AEs of the 30 mg group (I^2^ = 39%), which was mainly attributed to the Japanese subgroup study: the OR for AEs in the 30 mg group of this study was 12.32, significantly higher than that in other studies (OR range: 0.97-1.56). This is hypothesized to be related to the higher exposure to upadacitinib in the Japanese population, possibly due to a higher frequency of liver enzyme metabolism gene polymorphisms (e.g., CYP2C19*2/*3 genotypes) (22). To verify this impact, we excluded the Japanese subgroup study in the sensitivity analysis, which reduced heterogeneity to I^2^ = 21%. The overall conclusion remained unchanged (30 mg group: OR=1.35, P=0.03), further suggesting the robustness of the safety results in this study.

Publication bias analysis showed that the funnel plot for the ACR20 response rate in the 15 mg group versus the control group was symmetric (Egger’s test: P=0.38). This effectively reduced the bias of “publication of positive results being more likely”.

### 4.4 Study limitations and future research directions

While this systematic review adhered to the PRISMA guidelines, several limitations should be considered when interpreting the findings.

One important limitation lies in the scope and long-term assessment of key clinical endpoints. The present analysis focused primarily on the ACR20 response rate, while more stringent efficacy measures—such as ACR50 and ACR70 response rates—and composite disease activity scores, including the Disease Activity Score in 28 joints (DAS28), were not systematically analyzed. This restricts a more nuanced evaluation of the drug’s capacity to induce deep remission. In addition, the duration of treatment in the included trials was relatively short, with only two studies providing data up to 24 weeks. Consequently, a critical gap remains in long-term efficacy data beyond 52 weeks, particularly regarding inhibition of radiographic progression (e.g., assessed by the Sharp/van der Heijde score) and sustained improvements in patient-reported outcomes and quality of life (e.g., HAQ-DI and SF-36). Given that preventing structural damage is a fundamental treatment target in RA, the absence of long-term radiographic and functional data limits a comprehensive assessment of the long-term disease-modifying potential of upadacitinib.

Another key concern relates to insufficient population representativeness. The proportion of elderly patients (≥65 years) in the included studies was only 12%, and data on individuals with mild hepatic or renal impairment were lacking. As a result, evidence to support dose adjustment in special populations remains unavailable. This is particularly relevant in real-world clinical practice, where patients with RA frequently present with multiple comorbidities, underscoring the need for further evaluation of safety in these subgroups.

A further limitation is the inadequate exploration of dosing strategies. The present analysis included only two dosage regimens (15 mg and 30 mg) and did not incorporate data on sequential therapeutic approaches, such as dose de-escalation from 30 mg to 15 mg. Consequently, it is not possible to draw conclusions regarding the efficacy and safety of dose escalation following inadequate response to initial 15 mg therapy, nor to assess the feasibility of long-term maintenance treatment at lower doses.

In addition, the restriction to English and Chinese publications may have introduced language bias, potentially leading to the omission of relevant studies published in other languages.

In light of these limitations, future research should be directed toward several key areas. To begin with, long-term prospective real-world studies with follow-up durations of at least five years are warranted, particularly in East Asian populations. Such studies should incorporate comprehensive outcome measures, including long-term radiographic progression, physical function (e.g., HAQ-DI), health-related quality of life (e.g., SF-36), and rates of deep clinical remission (e.g., ACR50/70 and DAS28-defined remission). At the same time, systematic and rigorous monitoring of long-term safety outcomes—including major adverse cardiovascular events, venous thromboembolism, and malignancies—is essential to more precisely define the long-term benefit–risk profile of upadacitinib.

Equally important, dedicated randomized controlled trials focusing on special populations are needed. In particular, elderly patients (≥65 years) and individuals with mild hepatic or renal impairment should be systematically investigated to evaluate individualized dosing strategies, such as reduced starting doses (e.g., 10 mg), and to clarify optimal monitoring frequency and safety considerations in these subgroups.

Beyond this, future studies should explore sequential and optimization treatment strategies. This includes head-to-head comparisons between dose escalation approaches (e.g., increasing upadacitinib from 15 mg to 30 mg after inadequate response) and switching to biologic therapies. In addition, the efficacy and safety of combination strategies—such as upadacitinib 15 mg combined with short-course glucocorticoids for rapid disease control—should be evaluated to inform treatment adjustments following inadequate response.

Finally, efforts should be made to reduce potential language bias in future systematic reviews and meta-analyses. This may be achieved by including studies published in languages beyond English and Chinese through collaboration with multilingual researchers or the use of professional translation services. Expanding the search strategy to include regional and non-English databases, such as LILACS, SciELO, and other local repositories, may further improve study retrieval. Collectively, these approaches would enhance the comprehensiveness of evidence synthesis and improve the external validity and global applicability of the findings.

### 4.5 Clinical application recommendations and practical value

The present study contributes to the existing literature by specifically addressing dose-dependent differences between 15 mg and 30 mg upadacitinib, thereby providing a more refined understanding of its benefit–risk profile in rheumatoid arthritis. Based on the findings, upadacitinib 15 mg may be considered a preferred oral therapeutic option in selected patient populations.

In particular, it is suitable for patients with an inadequate response to conventional synthetic disease-modifying antirheumatic drug (csDMARD) monotherapy who decline injectable therapies, as the 15 mg dose demonstrates meaningful efficacy (ACR20 OR=4.09) while maintaining a relatively favorable safety profile, with a lower risk of overall adverse events (OR=1.29), thus achieving a balance among efficacy, safety, and treatment convenience. It may also serve as an alternative option for patients who are intolerant to biologic injections or who develop anti-drug antibodies, given its distinct mechanism of action. In addition, for patients with moderate-to-severe active rheumatoid arthritis requiring rapid symptom control, the 15 mg regimen may provide early clinical benefit, with differences in therapeutic response observable as early as week 1, thereby improving joint symptoms and quality of life. However, it should be emphasized that these comparisons are based on indirect evidence derived from different trial populations; therefore, conclusions regarding relative efficacy across JAK inhibitors should be interpreted with caution and ideally confirmed in future head-to-head randomized controlled trials.

In clinical practice, several key considerations should be emphasized. First, comprehensive risk screening is required prior to treatment initiation, including testing for hepatitis B surface antigen (HBsAg) and tuberculosis screening using either the tuberculin skin test (TST) or interferon-gamma release assays (IGRAs), in order to reduce the risk of reactivating latent infections. Second, dose selection should be individualized, with 15 mg generally preferred for patients without high-risk features such as advanced age, smoking history, or underlying cardiovascular disease, while escalation to 30 mg should be reserved for carefully selected patients with inadequate response and no contraindications. Third, structured monitoring is essential: during the first three months of therapy, complete blood counts (with particular attention to neutrophil levels) and liver function tests (ALT/AST) should be performed every four weeks, followed by every twelve weeks thereafter; in addition, annual assessment of cardiovascular risk factors, including blood pressure, lipid profile, and electrocardiography, is recommended to ensure treatment safety.

In conclusion, this meta-analysis suggests that upadacitinib 15 mg represents an oral therapeutic option for rheumatoid arthritis with a favorable balance of efficacy and safety. These findings provide evidence to support individualized treatment decision-making and contribute to the broader shift toward more precise and patient-centered oral therapeutic strategies in rheumatoid arthritis management.

## Data Availability

The original contributions presented in the study are included in the article/**Supplementary Material**. Further inquiries can be directed to the corresponding author.
