# Supplementary material for: Efficacy and safety of upadacitinib in the treatment of rheumatoid arthritis: a systematic review and meta-analysis
Source: Front Immunol. 2026 May 20;17:1806265. doi: 10.3389/fimmu.2026.1806265 (PMC13229858; doi:10.3389/fimmu.2026.1806265)
Supplement: Supplementary file 2 [file SupplementaryFile1.pdf]

Supplementary Material 1. Full Search Strategy

A comprehensive literature search was conducted to identify randomized controlled trials (RCTs) evaluating the efficacy and safety of upadacitinib in patients with rheumatoid arthritis. The search strategy combined controlled vocabulary (MeSH in PubMed and Emtree in Embase) with free-text terms. Field restrictions (Title/Abstract) were applied to improve specificity. No language restrictions were applied.

Databases Searched

PubMed
Embase

PubMed Search Strategy

("Arthritis, Rheumatoid"[Mesh] OR "Rheumatoid Arthritis"[Title/Abstract])

AND

("Upadacitinib"[Mesh] OR Upadacitinib[Title/Abstract] OR "ABT-494"[Title/Abstract])

AND

(randomized controlled trial[Publication Type] OR randomized[Title/Abstract] OR randomly[Title/Abstract] OR placebo[Title/Abstract])

Embase Search Strategy

('rheumatoid arthritis'/exp OR 'rheumatoid arthritis':ti,ab)

AND

('upadacitinib'/exp OR upadacitinib:ti,ab OR 'ABT-494':ti,ab)

AND

('randomized controlled trial'/exp OR random*:ti,ab OR placebo:ti,ab)

Additional Search Methods

Reference lists of included studies and relevant reviews were manually screened to identify additional eligible studies. Duplicate records were removed prior to screening.
